# Effects of Transcranial Direct Current Stimulation on Consolidation of Fear Memory

**DOI:** 10.3389/fpsyt.2013.00107

**Published:** 2013-09-24

**Authors:** Manish Asthana, Katharina Nueckel, Andreas Mühlberger, Dorothea Neueder, Thomas Polak, Katharina Domschke, Jürgen Deckert, Martin J. Herrmann

**Affiliations:** ^1^Department of Psychiatry, Psychosomatics and Psychotherapy, University of Würzburg, Würzburg, Germany; ^2^Department of Clinical Psychology and Psychotherapy, University of Regensburg, Regensburg, Germany

**Keywords:** transcranial direct current stimulation, dorsolateral prefrontal cortex, fear conditioning, fear memory consolidation

## Abstract

It has been shown that applying transcranial direct current stimulation (tDCS) over the dorsolateral prefrontal cortex (DLPFC) influences declarative memory processes. This study investigates the efficacy of tDCS on emotional memory consolidation, especially experimental fear conditioning. We applied an auditory fear-conditioning paradigm, in which two differently colored squares (blue and yellow) were presented as conditioned stimuli (CS) and an auditory stimulus as unconditioned stimulus (UCS). Sixty-nine participants were randomly assigned into three groups: anodal, cathodal, and sham stimulation. The participants of the two active groups (i.e., anodal and cathodal) received tDCS over the left DLPFC for 12 min after fear conditioning. The effect of fear conditioning and consolidation (24 h later) was measured by assessing the skin conductance response (SCR) to the CS. The results provide evidence that cathodal stimulation of the left DLPFC leads to an inhibitory effect on fear memory consolidation compared to anodal and sham stimulation, as indicated by decreased SCRs to CS+ presentation during extinction training at day 2. In conclusion, current work suggests that cathodal stimulation interferes with processes of fear memory consolidation.

## Introduction

Exaggerated fear is the root cause of the fear memory persistence, which further leads to the development of anxiety disorder (1, 2). A preponderance of neurobiological data from the past decades demonstrates that consolidation processes is responsible for making fear memories robust. Studies investigating the neurobiological nature of memory consolidation showed that memory consolidation is dependent on the interaction of neurons and their synapses (3–5). Hence, the process is commonly known as “synaptic consolidation” or “cellular consolidation”. Synaptic consolidation of memory highlights that synaptic plasticity plays an important role in learning and memory processes (4).

Research on learning and memory shows that the mechanism of synaptic plasticity depends upon several factors such as (i) initially activated neural circuit, (ii) release of second messengers, i.e., cyclic AMP (cAMP) and protein-kinase A (PKA), (iii) pre- and post-generated proteins, and (iv) the regulation of genes (4, 5).

In an *Aplysia* model, researchers have shown the changes occur during consolidation and formation process from short- and long-term (5). The incoming sensory inputs enhance the release of serotonin, which is followed by an increase of second messenger cAMP and PKA activity within the neuron. Furthermore, increase of PKA activity phosphorylates neurotransmitter channels, vesicles, and other proteins, which results in strengthening of the synaptic connections (5, 6). Schafe et al. (6) affirmed that protein synthesis is required during LTM storage, but not during STM storage. Protein synthesis during long-term storage strengthens the synaptic connections.

Furthermore, post-learning interferences of newly formed memory with electroconvulsive shock or protein synthesis blockers also modulate or disrupt LTM, but leave STM intact (7). It has been confirmed that the administration of propranolol, a beta-adrenergic receptor antagonist, during consolidation can disrupt this process (7–9).

Recent studies have shown that procedural and declarative memory consolidation can also be modulated by transcranial brain stimulation (10, 11). Elmer et al. (10) showed that cathodal transcranial direct current stimulation (tDCS) over the left dorsolateral prefrontal cortex (DLPFC) leads to decrease performance in short-term verbal learning compared with the placebo stimulation (sham) condition. Penolazzi et al. (11) demonstrated that tDCS could also modulate emotional memory. In detail, they reported that a combination of right anodal and left cathodal stimulation of the DLPFC facilitates the recall of pleasant compared to unpleasant and neutral images, whereas left anodal/right cathodal stimulation shows contrasting results. This double dissociation effect of tDCS adds evidence to the hypothesis of hemispheric specialization with regard to emotional processes.

Moreover, recent studies show that tDCS modulates attention (12), working memory processes (10, 13–15), and behavioral inhibition (i.e., difficulty to inhibit response) (16). Jacobson et al. (16) found that anodal tDCS to the right inferior parietal gyrus improves behavioral inhibition suggesting that tDCS modulates cognitive control in healthy individuals. Balconi and Vitaloni (17) demonstrated that cathodal tDCS over the left DLPFC and anodal tDCS over the right supraorbital area attenuates the cognitive load in the incongruent processing task and limits the incongruence effect generated by semantic anomaly.

Thus, converging evidence points to tDCS as a successful neuromodulator tool (43). Earlier findings robustly suggest tDCS interference with memory consolidation processes (10, 13–15). However, the neuromodulatory effect of tDCS on fear memory consolidation is still unknown, which is the focus of the current study. With respect to conditioned fear, we expect an enhancement of fear consolidation by anodal stimulation and attenuation by cathodal stimulation.

## Materials and Methods

### Participants

Sixty-nine students from the University of Würzburg were recruited through online advertisements to participate in the present study. The study was described in short, with the info that the study was conducted on 2 days, includes fear conditioning and a weak electrical stimulation of the brain. No psychology students were allowed. Participants received a financial gratification of 12 Euro in total on the second day. Participants were eligible for inclusion if they met the following criteria: (i) right handed; (ii) age 18–30 years; (iii) German native; (iv) females taking contraceptives during the study period. Participants were excluded from the trial if they met the following criteria: (i) any metal object or implant in brain, skull, scalp, or neck; (ii) implantable devices, including cardiac pacemakers and defibrillators; (iii) any neurological or psychiatric illnesses; (iv) pregnancy. Information about these criteria was obtained by questionnaires. Participants were randomly assigned into three groups: anodal, cathodal, and sham stimulation. The three stimulation groups do not differ with respect to the subjective ratings in anxiety sensitivity as measured by the ASI-3 questionnaire [anodal: 16.8 ± 9.1; cathodal: 16.2 ± 7.7; sham: 16.3 ± 9.8; *F*(2, 46) = 0.02; *p* = 0.98; ASI-3; (18)]. After data analysis (see below), three participants were excluded due to artifacts (i.e., origin of response to stimuli before a baseline in more than eight numbers of trials resulting in less than three artifact-free trials for each condition). Three subjects had to be excluded as they had only null responses; a null response was defined by amplitudes <0.01 μV. Seven participants were excluded because they do not show a conditioned response [conditioned stimuli (CS)+ > CS−during acquisition and also CS+ (acquisition)]. Two participants did not volunteer to participate on extinction training (24 h later: day 2). Three participants were rejected because tDCS stimulation stopped due to high-impedance of stimulation electrodes (>20 kΩ) (19). One participant was rejected as it was identified with depressive symptoms and one participant was recruited twice by mistake so only the first recorded data was considered in the current study. The demographical data of the remaining 49 participants is shown in Table 1. The study was in accordance with the declaration of Helsinki in their latest version from 2008 and has been approved by the ethics committee of the University of Würzburg. All participants gave written informed consent to participate in the study.

**Table 1:** **Demographic data of the participants**.

	Anodal tDCS	Cathodal tDCS	Sham
No. of participants	16	18	15
Age range	22.18 ± 2.26	23.11 ± 2.08	22.46 ± 2.38
Female/male	6/10	11/7	8/7

### Apparatus

Skin conductance responses (SCRs) were recorded by a constant voltage circuit with two 0.5 V across both electrodes and a 16-channel QuickAmp amplifier (Brain Products GmbH) with a sampling rate of 1,000 Hz and a notch filter of 50 Hz. Responses were recorded by using two Ag/AgCl electrodes (diameter = 13 mm) filled with non-hydrating gel. Both electrodes were attached to the volar surfaces on medial phalanges of the participants’ non-dominant palm (20). A digital display showed the skin conductance values, which were continuously recorded via Vision Recorder Version (1.0) software (Brain Products GmbH).

The unconditioned stimulus (UCS) consisted of 2000 ms of woman’s scream (code number 276) adapted from the International Affective Digital Sounds (IADS), delivered through an external sound card (Terratec, DMX 6 Fire USB) at an intensity of 102 db.

Colored squares (blue and yellow), serving as CSs, were presented at a 16° visual angle on central screen of 19″ monitor for 4 s with the inter-stimulus-interval (ISI) of 20–22 s and counterbalanced as CS+ and CS− to each participants, so that both squares were equally often selected as CS+ and CS−. The exposure time of the CSs and UCS was controlled using Presentation^®^ Version 13.0 software (Neurobehavioral Systems, Inc., Albany, CA, USA).

### Procedure

**Figure 1:**
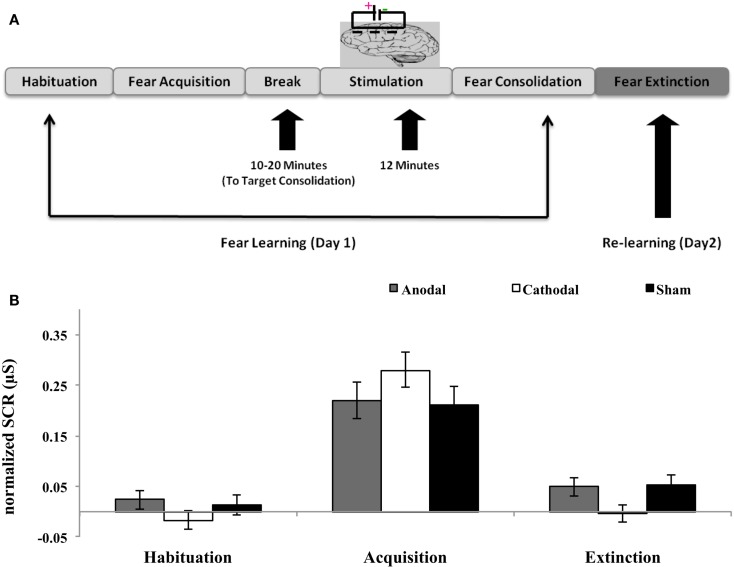
**Post-treatment of fear learning with tDCS disrupts fear consolidation**. **(A)** Schematic overview of experimental procedure. **(B)** Mean differential SCRs (CS+ minus CS−) during habituation, acquisition, and extinction (first five trials) for each experimental group (anodal, cathodal, and sham). The three groups showed normal fear acquisition. Fear return was observed in the group anodal and sham. In contrast there was no trace of fear observed in the cathodal group.

The present study was conducted at two consecutive days maintaining the time difference between two sessions from 20 to 26 h. The study involved three stages: habituation, acquisition, and extinction. The first session (day 1) consisted of the habituation and acquisition stages, in which participants learned the association of conditioned (CS) and unconditioned stimuli (UCS). On day 1, the experiment had two phases: (i) the habituation phase, consisting of eight trials in which squares of each color were presented on the monitor to reach a stable response to the stimuli and (ii) the acquisition phase, during which 16 trials of squares of each color were presented. During acquisition phase, one of the colored squares was paired with the scream (75% reinforcement rate); after 10–20 min of fear acquisition, electrodes for electrical stimulation were applied. As previous studies showed that the consolidation process occurs during the time window from minutes to 6 h (4, 21). Hence, in the current study all participants were stimulated after 10–20 min. The variable time frame of 10–20 min depended on the fitting of the tDCS electrodes. During the extinction phase (day 2), all participants underwent extinction training (see Figure 1). During extinction training, 16 CS+ and CS− were repeatedly presented in absence of the UCS.

### DC Stimulation

Transcranial direct current stimulation is a non-invasive method, in which direct current is applied over the scalp to modulate human brain excitability (22–24). The exact mechanism of tDCS is unclear, but it is assumed that several minutes of anodal tDCS excites the neurons below the stimulated region and leads to the depolarization, while cathodal tDCS has an inhibitory effect, i.e., it reduces the neuronal firing resulting in hyper-polarization. This suggests that tDCS functions via modulating the cortical excitability of the stimulated regions (25).

tDCS was delivered by a battery-driven stimulator (DC-Stimulator-Plus, NeuroConn GmbH, Ilmenau, Germany) approved for use in humans. A pair of conductive-rubber electrodes (size 5 cm × 7 cm = 35 cm^2^) coated with Ten20 cream conductive paste (Waever and Company, CO, USA) was positioned over the left DLPFC (electrode position F3) and left mastoid (reference electrode) (14, 26, 27) according to the international 10–20 electroencephalographic-system (28). For active tDCS, a constant current of 1 mA was applied for 12 min (current density: 0.0286 mA/cm^2^) (29, 30). The current was ramped up or down over the first and last 10 s of stimulation, respectively. The stimulator, always ranging below 20 kΩ, controlled the impedance. During sham condition the constant current was ramped up over the first 10 s once the DC had reached a current flow of 1 mA the current ramped down over 10 s. Therefore, the sham stimulation led to the same sensation in the participants, but had no long lasting effects (31, 32).

### Analysis

Participants were excluded from the analysis when not showing conditioning (CS+ < CS−during acquisition) and CS+ (acquisition) having more than 50% artifacts (i.e., ≥8 trials in CS+ or CS−) in the raw data of SCR. Participants were also excluded when the tDCS electrodes read impedances higher than 20 kΩ during the active and sham condition.

Offline analysis was performed in stepwise manner firstly recorded SCRs data was filtered at 1 Hz, and segmented into different phases (e.g., habituation, acquisition, and extinction) as well as single CS+ and CS− trials. Secondly, each segment was baseline corrected 1,000 ms prior to the onset of the stimuli and characterized by the peak response of the SCR signal between 1 and 4.5 s after stimulus onset. Finally, artifact rejection was conducted manually for all 72 trials per participants.

For the statistical analysis, the first CS+ and CS− trial were disregarded due to the orienting response at the beginning of the session in all phases (33). The remaining trials were used to produce average normalized SCR scores (CS+ and CS−) within subjects. Raw SCR scores were square root transformed to normalize distributions. To test the fear memory trace, after tDCS all trials of the acquisition phase (i.e., trials 2–16) were considered, however, to test the effect of tDCS on fear consolidation we looked at first five trials of the extinction phase (i.e., trials 2–6). It is worth mentioning that if consolidation is interfered, the analysis of early period of retention test hints the impairment of consolidation as suggested in literature of fear conditioning (33, 34).

The effect of stimulation on fear memory consolidation was analyzed by a three-factor ANOVA with stimuli (CS+ and CS−), time [acquisition (day 1) and extinction (day 2)], and group (anodal, cathodal, and sham) as main factors.

## Results

Means and standard deviations for SCRs in response to the conditioned stimulus are shown in Table 2. Two-way ANOVA was designed to assess differential fear response during habituation and acquisition among groups, with main factors of group (anodal, cathodal, and sham) and time (habituation and acquisition). The differential fear response was assessed by subtracting responses to the CS− from responses to the CS+ in corresponding trials. The differential scores were averaged across subjects. Statistic showed significant effect of time [*F*(1, 46) = 101.67; *p* < 0.01] but no effect of group or interaction [*F*(2, 46) = 2.57; *p* > 0.05]. This affirms that differential fear responses, i.e., SCR values did not differed significantly during habituation and acquisition among groups. Follow-up one-way ANOVA revealed non-significant differences during acquisition among groups [*F*(2, 46) = 1.42; *p* = 0.25].

**Table 2:** **Mean and SD of skin conductance response (μS) during fear-conditioning phases separately for the three tDCS groups**.

	Anodal tDCS		Cathodal tDCS		Sham	
	CS+	CS−	CS+	CS−	CS+	CS−
Habituation	0.10 (0.11)	0.08 (0.10)	0.06 (0.08)	0.08 (0.11)	0.05 (0.08)	0.04 (0.07)
Acquisition	0.33 (0.23)	0.11 (0.09)	0.38 (0.22)	0.09 (0.09)	0.28 (0.14)	0.07 (0.05)
Extinction	0.12 (0.17)	0.07 (0.10)	0.08 (0.09)	0.08 (0.10)	0.10 (0.14)	0.05 (0.09)

**Figure 2:**
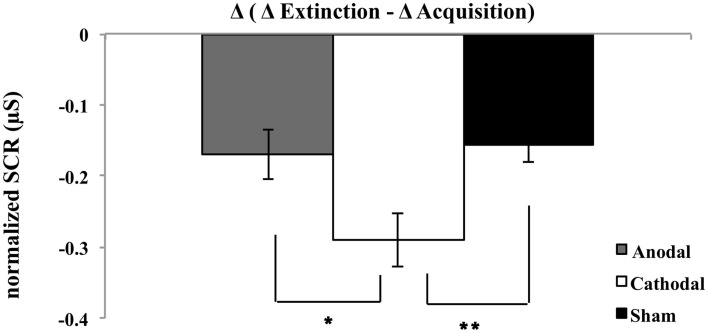
**Plot shows post-to-pre mean differential SCRs (Δ extinction minus Δ acquisition) for all experimental groups [i.e., anodal, cathodal, and sham]**. *P  < 0.05 and **P  < 0.01 (between acquisition and extinction); error bars represent standard errors.

To evaluate the effect of stimulation on fear memory consolidation, a three-factor ANOVA was calculated showing a significant main effect of stimuli [*F*(1, 46) = 106.32; *p* < 0.01], time [*F*(1, 46) = 32.73; *p* < 0.01], and interaction effects stimuli × time [*F*(1, 46) = 109.97; *p* < 0.01] and stimuli × time × group [*F*(2, 46) = 5.03; *p* = 0.01] (see Figure 2). A follow-up *t*-test was used to compare the anodal and cathodal group with regard to the amount of attenuation between acquisition and extinction phase for the differential values between CS+ and CS− (see Figure 2), which differed, significantly [*t*(1, 32) = 2.34; *p* < 0.05] with lower values for the cathodal group. In a similar way, the cathodal group also displayed diminished differential values compared to the sham group [*t*(1, 31) = −2.88; *p* < 0.01]. This might indicate that cathodal stimulation has an inhibitory effect on fear consolidation, while anodal stimulation and sham did not differ significantly from each other [*t*(1, 29) = −0.32; *p* > 0.05] (see Figure 2). Chi-square test revealed non-significant differences in the gender distribution among the three groups (anodal, cathodal, and sham) (χ^2^ = 1.94, *df* = 2, *p* = 0.38).

## Discussion

To investigate the effects of tDCS on the fear consolidation process, we applied left DLPFC stimulation to healthy participants for duration of 12 min after fear conditioning. The results show that cathodal, but not anodal stimulation disrupts fear memory consolidation.

The DLPFC has an important role in memory and learning processes (10, 13). Studies using a verbal-memory task (10) or N-back task (13), respectively, showed improvement in memory after stimulating the left DLPFC, indicating the efficiency of tDCS in modulating DLPFC activity (14, 19, 35). Moreover, improvement in memory was due to anodal stimulation of the DLPFC. However, cathodal stimulation showed a reciprocal effect (35). In an EEG study, Zaehle and colleagues observed reduced accuracy in a working memory task along with tDCS of the DLPFC. The main result of the present study was an impaired fear memory consolidation after left DLPFC stimulation, which further supports the role of DLPFC stimulation in memory consolidation (10, 11, 13–15, 36). In synopsis of evidence from the existing literature and the present results show a crucial impact of tDCS over the left DLPFC on fear memory consolidation processes.

Baudweig et al. (37) demonstrated in a neuroimaging study, that 5-min of cathodal tDCS leads to 38% reduction in cortical excitability, while 15–20 min of stimulation leads to a reduction of 28%. In contrast, they failed to observe any changes in cortical excitability after anodal tDCS. Nevertheless, their findings demonstrated the efficacy of tDCS in the modulation of cortical excitability in humans. Moreover, recent neuroimaging study by Keeser et al. (38) showed the modulation of resting state functional connectivity via prefrontal tDCS. They well demonstrated in their study that the blood oxygen level dependent (BOLD) Magnetic Resonance Imaging (MRI) has enhanced neural activations between frontal brains regions and tDCS applied brain regions.

Moreover, studies investigating sleep and memory processes showed that the spindle activity generated during slow-wave-sleep (SWS) is important in sending information to the hippocampus, which acts as a buffer and stores the relevant information. The generated spindle activity during SWS activates the newly encoded information in the hippocampus resulting in consolidation. This reactivation of hippocampal memory via spindle activity synchronizes with the neo-cortex and sends the newly stored information back to the neo-cortex which further triggers LTP processes (39). The spindle activity assists the strengthening of neuronal connections and potentiates consolidation. The induction of DC stimulation on the frontal area (F3) of the brain might facilitate the slow oscillatory activity, which has been suggested to aid in the potentiation of consolidation processes (14).

Lin et al. (40) showed that fear acquisition and fear extinction share some common and uncommon mechanism. They suggested that both processes require protein synthesis (cAMP response element-binding protein), kinase (phosphatidylinositol 3-kinase), and NMDA receptors in the amygdala for the consolidation mechanism. Furthermore, they demonstrated long-term behavioral changes after fear and its extinction. It was argued that learning about fear as well as extinction triggers a Ca^2+^ ion influx in amygdalar neurons. The increase in Ca^2+^ ion concentration promotes long-term synaptic depression (LTD) (14, 40). This suggests that extinction processes suppress Ca^2+^ ion concentrations and lead to synaptic changes, which may lead to extinction of fear memory.

It is important to address here that the tDCS parameters used in the current work ensure better control to investigate its efficacy in conditioning fear. Nitsche and Paulus (29, 30) affirmed earlier that an electrical stimulation lasting 10–13 min modulates cortical excitability and shows long-term effects up to 90 min.

Since slight modification via induction of low potential negative or positive potential might be sufficient to modulate mental processing effectively, neural connectivity and excitability holds a sensitive mechanism (44). In addition, the higher the amplification of induced current the more effective it would be, but the exploration of tDCS effects in psychiatry disorder has not yet been completed. With our findings we propose that the amplification used in the current work would be sufficient and effective in similar study and paradigm.

One point we have to discuss is the number of drop outs in our sample. Ten out of 69 were rejected by the fact that the conditioning procedure was not working. The number of 19 dropouts out of 69 participants is similar to other studies using a fear-conditioning paradigm [for example (41), with a 23% drop out rate, compared to 27% dropout rate in our study]. However the study by Glenn and colleagues also found that using a scream as UCS like in our study is efficient, but an electric shock is more aversive leading to a better conditioning effect, which might be the reason for our high dropout rate. A second point which should be discussed is the fact, that our participants were not less sensitive for anxiety symptoms (mean value 16.3) as assessed with the ASI-3 questionnaire, compared to other healthy populations [for example (42): 13.8 in college students but 25.2 and above in different anxiety patient samples]. It might be that this low ASI-3 values also contribute to high dropout rate to missing conditioning. In summary, we do not think that this dropout rate was higher compared to other studies, especially as we had some additional factors leading to addition drop out, like measuring on two consecutive days, and using tDCS, which also lead independently to drop outs.

Although, the current study demonstrated the efficacy of tDCS over left DLPFC on fear memory consolidation, some limitations of this finding must be taken into account. Firstly, methodological limitation of tDCS protocol and the modulation of left DLPFC response to certain stimulation reflect a narrow perspective of functional connectivity and cortical excitability. Secondly, due to limited sample size, the results might be biased with the number of random events. Finally, we also make no claim, whether tDCS influenced the fear association or fear recall.

In conclusion, our findings highlight that the application of cathodal tDCS over left DLPFC disrupts fear memory consolidation. However, anodal stimulation has no enhancing effect on conditioned fear. Moreover, the presently suggested role of the DLPFC in extinction and in turn the negative effects of cathodal tDCS on fear memory may contribute to novel therapeutic approaches in the prevention of the development of pathological memories, i.e., PTSD.

## Conflict of Interest Statement

The authors declare that the research was conducted in the absence of any commercial or financial relationships that could be construed as a potential conflict of interest.
